# Introduction of Temperature-Gradient Elution in Three-Dimensional Correlation Thermal Field-Flow Fractionation with Quintuple Detection for Monitoring Compositional Dynamics of Ultrahigh-Molar-Mass Poly(styrene-*co*-maleic anhydride)

**DOI:** 10.1021/acsmacrolett.5c00593

**Published:** 2025-12-16

**Authors:** Upenyu L. Muza, Michael-Phillip Smith, Joshua T. Johani, Bert Klumperman, Albena Lederer

**Affiliations:** † Department Advanced Macromolecular Structure Analysis, 28408 Leibniz-Institut für Polymerforschung Dresden e.V., Hohe Str. 6, D-01069 Dresden, Germany; ‡ 26697 Stellenbosch University, Department of Chemistry and Polymer Science, Private Bag X1, Stellenbosch 7599, South Africa

## Abstract

Analogous to temperature-gradient interaction chromatography, temperature-gradient elution three-dimensional correlation thermal field-flow fractionation (TGE-3DCoTF3) coupled with quintuple-detection provides high-resolution analysis of ultrahigh-molar-mass poly­(styrene-*co*-maleic anhydride) synthesized via biradical photoinitiation. TGE-3DCoTF3 resolves more than 3 orders of magnitude in molar-mass, cleanly separating residual monomers and oligomers from megadalton copolymers while preserving their integrity. Multidetector correlation yields molar-mass, three independent radii, intrinsic viscosity, diffusion coefficient, thermal diffusion coefficient (*D*_T_) and UV–vis spectra. Differential UV–vis absorptivity distinguishes unreacted monomers from copolymers, enabling conversion analysis. *D*_T_ serves as a pseudospectroscopic probe, confirming constant styrene-to-maleic anhydride ratios and truly alternating copolymerization. Comparative analysis of “lower” and “higher” ultrahigh-molar-mass copolymers differing by a factor of 2 in degrees of polymerization reveals identical thermophoretic behavior but distinct coil compactness, evidencing transitions toward denser core–shell morphology. Collectively, TGE-3DCoTF3 offers a nondestructive, multidimensional benchmark for elucidating molar-mass, size, and compositional dynamics in polymers that challenge conventional SEC characterization.

## Graphical abstract

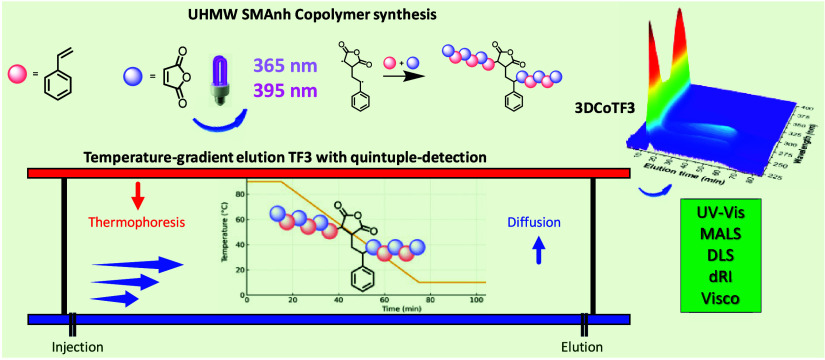

Poly­(styrene-*co*-maleic anhydride) (SMAnh) readily attains ultrahigh molar-masses (UHMW)­s above 10^5^ g·mol^–1^ through photoexcited charge-transfer complexation between styrene and maleic anhydride.^1^ These linear copolymers can exhibit exceptional mechanical and thermal stability, yet their size and fragility render conventional size-exclusion chromatography (SEC) unreliable due to shear degradation, electrostatic interactions, and coelution of species with similar hydrodynamic radii (*R*_H_) but different compositions.^2^_’_ ^3^ SEC performance further deteriorates above 10^7^ g·mol^–1^ because of polymer-pore anchoring and viscous drag, emphasizing the need for alternative separation methods.^4^

Thermal field-flow fractionation (TF3) circumvents these limitations by separating macromolecules in an open channel under a perpendicular temperature gradient.^5^ Thermophoresis drives solutes toward the cold wall according to their chemical composition, quantified by the thermal diffusion coefficient (*D*_T_).^6^ The steady-state elevation of macromolecules is governed by the Soret coefficient (*S*_T_), which depends on chemical composition and microstructure, but is largely independent of molar-mass.^7^ Random Brownian motion adds size selectivity, enabling concurrent fractionation by both size and composition, crucial for SMAnh copolymers where styrene- and maleic-anhydride-rich segments differ in thermophoretic behavior.^8^ Without a stationary phase, TF3 achieves nearly 100% sample recovery and preserves fragile UHMW species that degrade under SEC or high cross-flow asymmetrical-flow field-flow fractionation (AF4).^9^ TF3 thus provides a nondestructive method for characterizing UHMW SMAnh and even charged macromolecules.^10^ Although *D*_T_ is a complex parameter, it remains highly sensitive to small variations in composition and microstructure,^11,12^ giving TF3 unique selectivity for copolymers,^13^ microstructure gradients,^5^ and even cross-linked networks.^14^

Coupling TF3 to quintuple-detection enables simultaneous access to molar-mass, radius of gyration (*R*_G_), *R*_H_, intrinsic viscosity ([η]), concentration, composition (*D*_T_) and diffusion coefficient (*D*) in a single run.^15^ The evolution from quadruple-to-quintuple-detection is state-of-the-art in advanced analysis of complex polymers.^15,16^ Quintuple-detection is a modular system composed of five physical detectors, namely, multiangle light scattering (MALS), dynamic light scattering (DLS), viscometer (Visco), differential refractive index (dRI), and UV–vis. Notably, UV–vis doubles up as a concentration detector and a spectrophotometer for compositional analysis.^17^ Introducing a controlled temperature-program converts TF3 into temperature-gradient-elution three-dimensional correlation TF3 (TGE-3DCoTF3), extending the concept of temperature-gradient interaction chromatography (TGIC). Each elution slice becomes a node in a multidimensional data set interlinking retention (size/composition), optical contrast (e.g., UV–vis), and physicochemical parameters.

By cross-correlating these multiple-dimensions, TGE-3DCoTF3 converts the raw detector streams into high-resolution landscapes of molar-mass, size, microstructure and compositional heterogeneity in a single experiment.^18^ Preliminary aspects of this conceptual leap in data correlation was first demonstrated strictly on metallic nanostructures, where time-resolved UV–vis spectra (capturing the plasmonic resonance of embedded gold nanoparticles) were correlated with TF3 retention time (*t*_R_) to disentangle overlapping distributions in particle size, shape and gold content.^19^ The three-dimensional treatment delivered sensitivities and peak capacities far more descriptive than conventional TF3-UV–vis hyphenations.^17^ More importantly, this framework is not tied strictly to plasmonic systems; any spectroscopic contrast that varies across the separation window can populate the second axis.^20^

Overall, TGE-3DCoTF3 maps UHMW SMAnh fractionation in real-time, tracking monomer conversion, molar-mass distribution, and coil expansion via five orthogonal detectors. The method delivers cross-correlated physicochemical insights with intrinsic UV–vis spectral contrast and minimal coil interaction, extending classical TF3 into a multidimensional platform beyond the limits of SEC and AF4.

## Methods

### Biradical Initiation Photoinitiated Copolymerization

^1^ See Scheme 1 and SI Scheme S1: Further details on the copolymerization are furnished in SI Section II. A final sample was collected at the end of the polymerization and utilized to determine monomer conversion, and a parallel control experiment was performed with styrene alone (0.50 g, 4.80 mmol) under identical conditions.

**Scheme 1:**
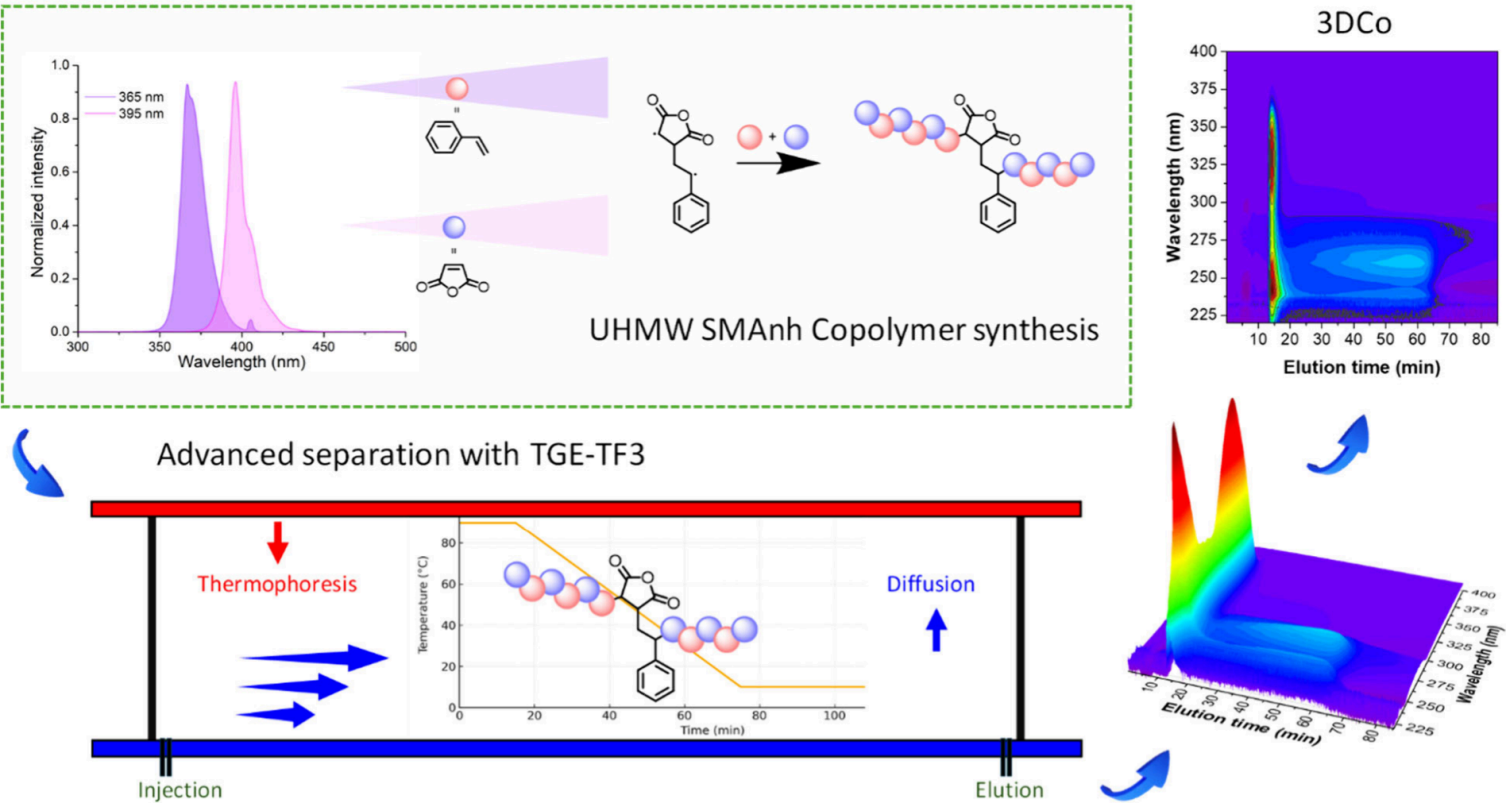
Advanced Separation Using TGE-3DCoTF3: UHMW SMAnh from the Biradical Initiation in Photoinitiated Copolymerization of Styrene and Maleic Anhydride^a^

a Initiation occurs via the excitation of a charge transfer complex with 365 or 395 nm light sources to generate a biradical species, and polymer chain growth; subsequently, the analytical process after injection to elution as a function of thermophoretic- and diffusion-induced mass-transfer under a controlled temperature gradient.

### TGE-3DCoTF3 of UHMW SMAnh

The 3DCoTF3 with quintuple-detection system used is outlined in detail in SI, Section S1.1, using tetrahydrofuran (THF) as a carrier solvent for all analyses. TF3 separations were executed with an initial Δ*T* = 90 °C (Scheme 1). For the linear drop in wall temperature from *T*_0_ = 90 °C to *T*_f_ = 10 °C, over a programmed-time interval Δ*t* (60 min), the temperature profile is fully described by eq 1:

1 $$T\left(t\right)=T_{0}-\frac{T_{0}{-T}_{f}}{\Delta t}t,0\leq t\leq \Delta t$$

### Comparative SEC Results

Due to the megadalton molar-mass range associated with this work, SEC results were not reliable or reproducible due to column interactions and none-SEC elution modes (Figure S3 and Table S2), and this constitutes the basis for exploring TF3 as an alternative.

TGE-3DCoTF3 with quintuple-detection fuses TF3 separation with time-resolved multiple-wavelength UV–vis spectroscopy, so that every point in the elution profile is linked to a full optical spectrum, allowing size-, shape- and composition-dependent distributions of complex and heterogeneous polymer systems to be mapped simultaneously with markedly higher resolution than conventional TF3 or batch UV–vis.^19^ Two strategic types of UHMW copolymers were considered for establishing a proof-of-concept: (i) lower UHMW and (ii) higher UHMW, with the latter having double the degree of copolymerization, relatively. Quintuple-detection elution profiles at 0.2 mL·min^–1^ flow rate formulate the main quantitative analytical discourse (Figure S1). However, higher flow rates (0.3 mL·min^–1^) were preferred for generating qualitative UV–vis spectral data to decrease *t*_R_ and consequently condense the overall data (Figure 1).

**Figure 1:**
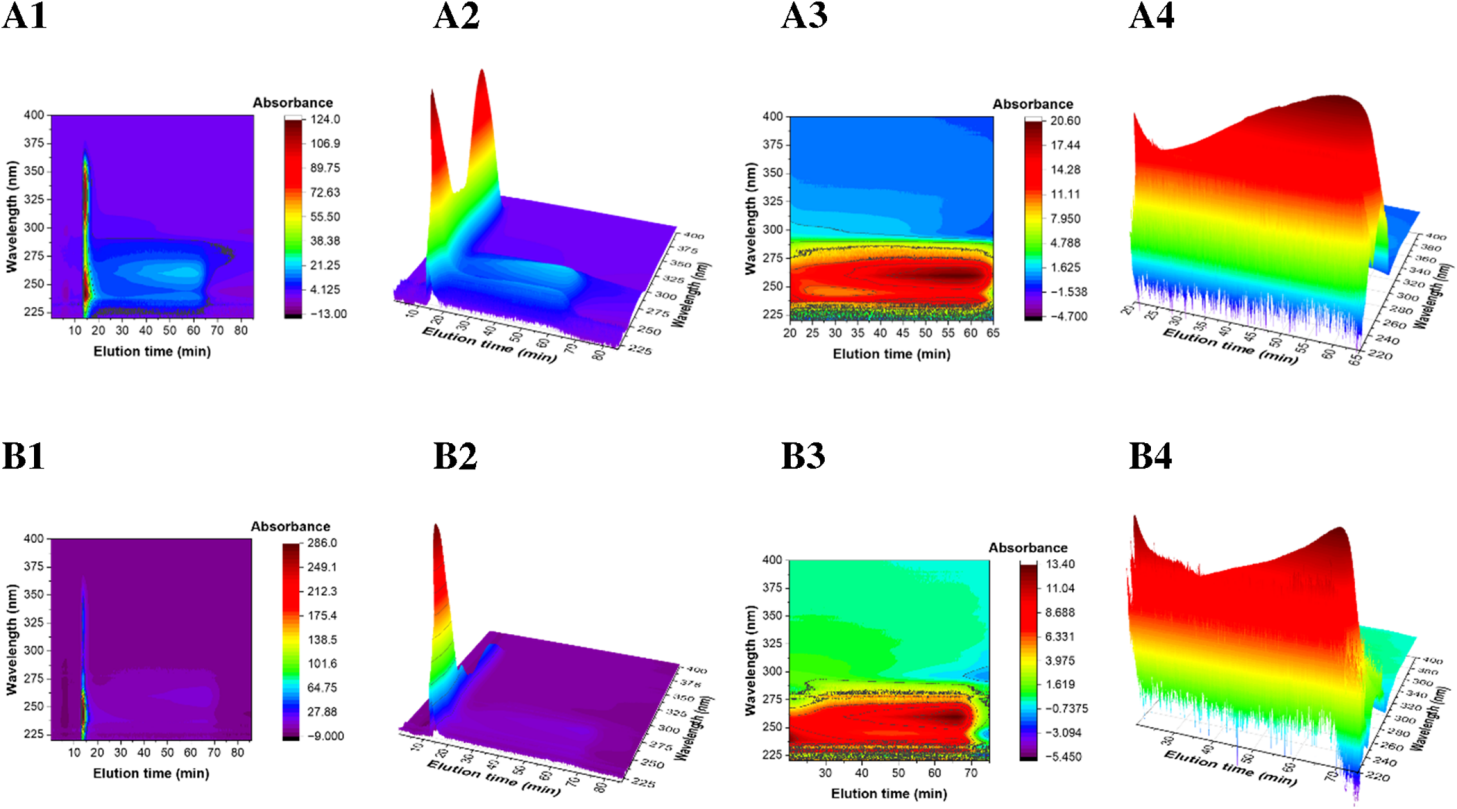
TGE-3DCoTF3 of SMAnh at (A) lower UHMW and (B) higher UHMW, using 0.3 mL·min^–1^ flow-rate; (A1 and B1) are UV–vis 2D/Contour plots and (A2 and B2) are corresponding 3D plots for SMAnh copolymerization, highlighting a deceptively intense *t*_0_ ≈ 15 min trace due to high molar-absorptivity of styrene (254 nm) and maleic anhydride (340 nm) monomers, enhanced compression of the main copolymer peak, despite >95% NMR-confirmed conversion; The main retention regions (*t*_R_ ≈ 20–75 min) shows stratified UHMW copolymer distribution via thermophoresis and diffusion, with UV activity at 254 nm indicating covalent bonding, although limited by insufficient signal-to-noise ratios for low-molar-mass quantification; (A3 and A4, and B3 and B4) are the corresponding zoomed-in spectra across the main peak (≈*t*_R_ 20–65/75 min, respectively).

TGE-3DCoTF3 offers detailed size and composition-resolved snapshots of SMAnh, and in particular, explains why the UV–vis trace at *t*_0_ is deceptively intense (Figure 1) despite overall monomer conversion in excess of 95%, as confirmed by NMR. Similar trends are observed for both lower and higher UHMW copolymers. Relative to the main copolymer peaks (*t*_R_ ≈ 20–65/75 min for lower and higher UHMW copolymers, respectively); there is intense UV–vis absorbance in the regions of both St (254 nm) and MAnh (340 nm) along *t*_0_ ≈ 15 min which misleadingly implies a copolymerization mechanism with low monomer conversions. The UV–vis absorbances at *t*_0_ are strikingly large for two mutually reinforcing reasons. First, molar absorptivity: both monomers retain their conjugated C=C double bonds, giving extinction coefficients at 254 and 340 nm (Figure S2) that are an order of magnitude higher than those of the corresponding repeat units in the copolymers, whose π-systems are disrupted during polymerization. Trace amounts of residual monomers therefore produce disproportionately strong optical signals. Second, peak compressions: nonretained molecules travel through the channel as a single, narrow band and reach the detector almost simultaneously; therefore, local concentration in the flow-cell is far higher than if the same masses were spread over a broader elution window. The combination of high molar-absorptivity and temporal compression exaggerates the UV–vis intensity without contradicting the NMR determined conversions.

Figure 1 shows that beyond the *t*_R_ ≈ 20 min limit in general, the fractograms rise into the main retention regions. Here, thermophoresis and diffusion balance to stratify macromolecules according to the product *S*_T_·Δ*T* and *R*_H_ or *D*. Generic copolymer UV–vis activity in the range *t*_R_ ≈ 20–75 min is confined to 254 nm, confirming that the maleic-anhydride chromophore is now covalently bound. However, insufficient signal-to-noise ratios of the concentration detectors (dRI and UV–vis) and MALS (Figure S1) prevents reliable quantification of this low-molar-mass fraction.

For quantitative analysis, a more stable and gentler separation is preferred, as such, the flow-rate was reduced by a third to 0.2 mL·min^–1^ to minimize pressure effects. Despite increasing *t*_R_, this adjustment was overly inconsequential (Figure S1a). Furthermore, given the plausible core–shell morphology/effects in higher UHMW polymers (as shall be discussed further),^21,22^ the more reliable data from lower UHMW sample dominates our main arguments. For further introspection, the lower UHMW elution profile is split into 3 strategic fractions (F), see Table 1 and Figure S1b. These 3 fractions provide explicit leverage for probing how integration limits control the reported averages. However, molar-mass results from a wider integration range are reported in SI, Table S2, as they provide more accurate representation of the expected *Đ* (≥1.5) from radical polymerization. F3 “maximum” fraction isolates the apex of the peak (*t*_R_ ≈ 77–83 min), and therefore, zooms into the highest weight-average molar mass (*M*_W_), mass concentration, lowest dispersity, and the greatest *R*_G_/*R*_H_ values – ideal for confirming UHMW copolymer tail. F2 is the “narrow” window (*t*_R_ ≈ 65–85 min), capturing the central body of the peak by trimming off most early low-*M*_W_ material but keeping the late elution band. F1, the “broad” window (*t*_R_ ≈ 52–85 min) extends well into the early eluting shoulder where smaller species elute, and therefore gives the lowest *M*_W_, the widest size distribution and highest dispersity, and is best suitable for complete mass-balance checks or when cumulative averages over the whole molar-mass range are needed. However, low signal-to-noise ratios negatively affect data processability in the lower concentration and lower molar-mass regions (Figure S1b).

**Table 1:** Results from TGE-3DCoTF3 with Quintuple-Detection of 3 Critical Fractions (F) for Two Copolymers at (A) Lower UHMW and (B) Higher UHMW, where B is Double the Molar Mass of A

A	lower UHMW copolymer													
F	Δ*T* (K)	*t*_R_ (min)	*D*_T_ × 10^–7^ cm^2^ (s/K)	*S*_T_ (K^–1^)	*M*_w_ (kg/mol)	*Đ*	*R*_G_ (nm)	*R*_H_ (nm)	*R*_V_ (nm)	α	*N*	ρ	[η] (mL/g)	*D* × 10^–7^ cm^2^ (s)
1	18.7	66	4.32	1.4	910	1.3	28	17	25	0.70	0.67	1.65	117	3.2
2	10	75	8.35	2.8	1116	1.1	31	18	29	0.76	0.64	1.71	135	3.0
3	10	80	8.06	3.0	1360	1.0	35	20	33	0.84	0.63	1.75	157	2.7

B	higher UHMW copolymer													
F	Δ*T* (K)	*t*_R_ (min)	*D*_T_ × 10^–7^ cm^2^ (s/K)	*S*_T_ (K^–1^)	*M*_w_ (kg/mol)	*Đ*	*R*_G_ (nm)	*R*_H_ (nm)	*R*_V_ (nm)	α	ν	ρ	[η] mL/g	*D* × 10^–7^ cm^2^ (s)
1	18.7	66	4.40	1.3	1705	1.4	42	17	33	0.67	0.41	2.47	135	3.4
2	10	75	8.08	2.8	2090	1.1	47	19	37	0.73	0.26	2.47	157	2.9
3	10	80	7.46	2.99	2570	1.0	50	22	42	0.85	0.10	2.27	183	2.5

For further introspection on the higher UHMW, Table 1B shows that doubling the molar-mass does not significantly alter *S*_T_ and *D*_T_ over the same F time-windows as lower UHMW (Table 1A), suggesting thermophoresis depends more on chain architecture and solvation. Figure S1c presents the MALS fractogram of the higher-UHMW copolymer, while Figure S1d overlays the lower- and higher-UHMW traces, clearly showing the expected shift to longer retention for the higher-UHMW sample. The core–shell theory provides a robust explanation for the unusually low conformation exponents (0.10–0.40) in the higher-UHMW copolymer.^21,22^ It suggests that beyond a critical molar-mass, additional polymer segments contribute primarily to a dense, poorly solvated core rather than to coil expansion, causing the scaling between size and mass to flatten. Thus, the core–shell structure effectively decouples mass growth from hydrodynamic expansion, leading to the low apparent conformation exponents observed. This indicates a morphological transition from an open random coil to a compact, hierarchical macromolecular assembly, a key insight that the multidetector approach captures; however, this requires validation with X-ray/neutron scattering.

The comparative results for lower and higher UHMW copolymers clearly demonstrate how polymer coils can exhibit identical *R*_H_ while differing in viscosity radius (*R*_V_) and molar-mass. Despite both copolymers showing comparable diffusion-derived *R*_H_, the higher UHMW species possessed nearly twice the molar-mass and a noticeably larger viscosity radius and intrinsic viscosity. These findings underscore that *R*_H_ alone cannot fully describe macromolecular conformation, as it reflects only translational diffusion, whereas *R*_V_ and [η] are sensitive to the internal density and topology of the polymer coil. The results thus provide direct experimental evidence that polymers with the same hydrodynamic size can differ substantially in molecular weight and coil architecture, emphasizing the complementary nature of the multidetector approach for comprehensive macromolecular characterization.

In Table 1A, the measured physicochemical descriptors for fractions F1 through F3 paint a coherent picture of progressive increase in the degree of polymerization. As expected, *M*_W_ increases from 9.1 × 10^5^ g·mol^–1^ in F1 to 1.36 × 10^6^ g·mol^–1^ in F3, while the dispersity decreases from *Đ* = 1.3 to 1.0, showing that the later-eluting material is not only heavier on average but also markedly narrower in size distribution. Nevertheless, the trend in *Đ* is generally anticipated when analyzing broader-narrower fractions of an elution profile. Concomitantly, the size metrics extracted from light-scattering and intrinsic-viscosity detection follow the same monotonic trend: *R*_G_ grows from 28 to 35 nm, *R*_H_ from 17 to 20 nm, *R*_V_ from 25 to 33 nm (Figure 2a), and [η] rises from 117 mL·g^–1^ to 157 mL·g^–1^ (Figure 2g). These increases are mirrored by a steady fall in the diffusion coefficient, from 3.2 × 10^–7^ cm^2^·s^–1^ for F1 to 2.7 × 10^–7^ cm^2^·s^–1^ for F3, consistent with Stokes–Einstein expectations for successively larger coils. Similar trends are observed in Table 1B, notwithstanding the limitations imposed by the core–shell effect. Nota bene, when calculating *D*_T_ using eq 2, unavoidably, Δ*T* across the channel prompts nonuniformity in viscosity, thus falsifying the description of the retention ratio (*R*), as defined by eq 3.^23^ However, both eqs 2 and 3 remain valid for our specific context, given that the highest Δ*T* (18.7 K) is low at subroom temperature; and accordingly, insufficient for significant distortions to the viscosities in question.

2 $$D_{T}=\frac{D}{\lambda \Delta T}$$

3 $$R=\frac{t_{0}}{t_{R}}=6\lambda \left(\coth\left(\frac{1}{2\lambda }\right)-2\lambda \right)$$

**Figure 2:**
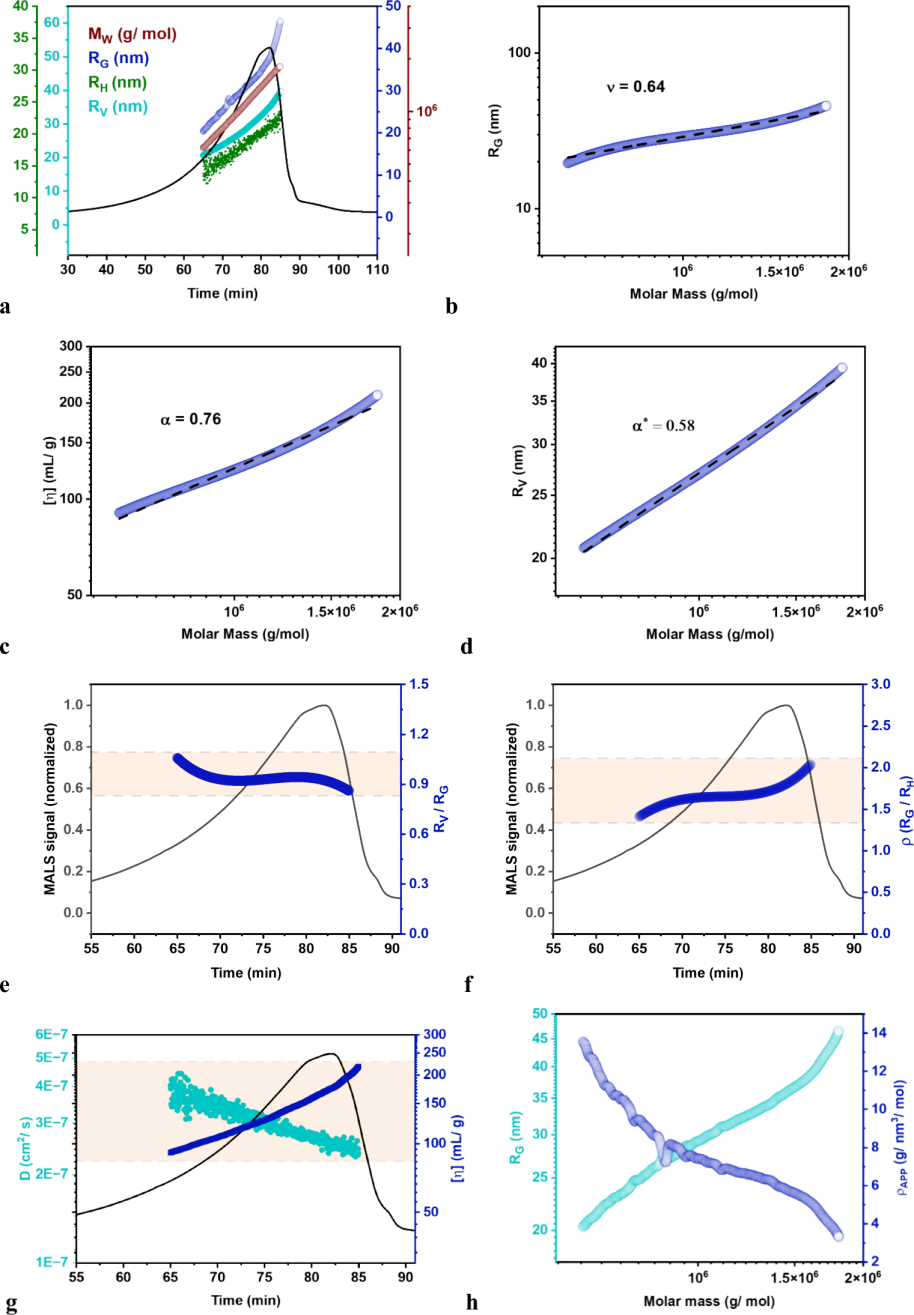
Comprehensive TGE-3DCoTF3 with quintuple-detection analysis of lower UHMW SMAnh copolymers. (a) Fractogram (black line, MALS signal) with online detector responses showing weight-average molar mass *M*_W_ (brown, right axis), *R*_G_ (blue), hydrodynamic radius *R*_H_ (green), and viscometric radius *R*_V_ (turquoise) as functions of retention time. (b) Double-log plot of *R*_G_ vs *M*_W_ with conformation exponent ν = 0.64. (c) MHS plot of intrinsic viscosity [η] vs *M*_W_ with exponent α = 0.76. (d) *R*_V_ vs *M*_w_ on double-log scales, yielding α* = 0.58; Evolution of chain-conformation descriptors in the main TF3 elution band (peach shading). (e) Time-resolved compaction factor, the ratio of the viscometric radius to the root-mean-square radius (*R*_V_/*R*_G_, blue, right axis), superimposed on the normalized MALS fractogram (black, left axis). (f) Shape factor ρ = *R*_G_/*R*_H_ (blue, right axis) across the same time window, rising from ≈1.4 to ≈2.0 as the coils become more compact. (g) DLS measured diffusion coefficient *D* (turquoise, left axis) and intrinsic viscosity [η] (violet, right axis) vs retention time; (h) Mass-dependent correlations extracted from the shaded slice: *R*_G_ (turquoise, left axis) and coil-density/shape parameter ρ (blue, right axis, units g·nm^–3^·mol^–1^).

To explain the separation trends as a function of *S*_T_: In general, fractions that diffuse slower (larger size) show a higher *S*_T_, consistent with TF3 predictions that *S*_T_ rises with coil size/molar-mass for flexible polymers. Later-eluting slices have higher *S*_T_; under a given program this is exactly what the TF3 retention equation anticipates because *S*_T_ drives exponential concentration bands closer to the cold wall, thus lowering their average flow velocity.^24^ However, *S*_T_ is not only dependent on the translational diffusion *D*, which corresponds to the hydrodynamic size, but also on the thermophoretic properties which are influenced by the chemical composition or molecular structure and is expressed in the behavior of *D*_T_.

Bearing in mind that *D*_T_ is a quantitative measure of chemical composition: F1 elutes when Δ*T* is decaying, therefore, its higher ΔT should generally give lower *D*_T_ (∼3 × 10^–7^ cm^2^·s^–1^·K^–1^), as shown in Table 1. F2 and F3 experience the minimum programmed gradient (10 °C), hence, their *D*_T_ values look almost doubled relative to F1 despite close similarities in terms of *D* (and *R*_H_). This doubling is rather expected, given the temperature-program used for fractionation results in differences in Δ*T* between F1 vs F2 and F3 by a factor of 1.87. Therefore, at Δ*T* 10 K, F1 in Table 1A is expected to have a *D*_T_ of ∼8.08 × 10^–7^ cm^2^·s^–1^·K^–1^, and remarkably, this closely resembles that of F2 and F3. This implies that there is no significant heterogeneity in composition from F1 to F3, and thus, the comonomer ratios remain constant across the given *M*_w_ range.^25^ Therefore, the copolymerization conforms to statistically uniform composition during chain growth, as both St and MAnh are incorporated in a consistent alternating mechanism, regardless of the degree of polymerization.^26^ This highlights the unique application of *D*_T_ as a sensitive probe to chemical structure similar to spectroscopic techniques such as FTIR, NMR, for monitoring the compositional integrity of the copolymer during chain growth. The slight decrease from F2 to F3 mirrors their moderate decrease in *D*, a trend often seen as size increases; and as expected, chain entanglements minimize size increase with the degree of copolymerization.

Several dimensionless shape parameters corroborate the growth in segment density that accompanies the mass increase (Figure 2h), and the average values over the fractions are more reliable for lower UHMW (Table 1A). Notably, the fractions are overlapping, and therefore, the shape parameters are averages over variable molar mass ranges. The MHS exponent α (ratio of the observed intrinsic viscosity to the ideal Gaussian-coil value) rises from 0.70 to 0.84, while the ratio ρ = *R*_G_/*R*_H_ ascends slightly from 1.65 to 1.75, values typical of random-coil polymers yet edging upward as coils become more expanded at UHMW. The conformation exponent ν, drifts downward from 0.67 toward 0.63, indicating that the later fractions approach the asymptotic statistical coil in a good-solvent value (0.59) where hydrodynamic shielding is more complete.^16,27^

Taken together, these coupled trends confirm that fractionation by TF3 effectively sorts the SMAnh according to coil size and mass, delivering progressively purer UHMW populations with minimal tailing of lower-mass material in the densest window (fraction F3). The most reliable introspection on property distributions is based on F2 at *t*_R_ ≈ 40–70 min (Table 1 and Figure 2). Quintuple-detection data yields *M*_W_ of 1.12 × 10^6^ g·mol^–1^ with a low *Đ* of 1.1 (Table 1A). However, as previously mentioned, higher dispersities (≥1.5) are reported across the more extended elution profile, consistent with radical polymerization mechanisms. Regardless, the molar-mass distribution across the peak spans over an order of magnitude and is sufficient to obtain reliable dependencies of both [η] and *R*_G_ on molar-mass, and hence more reliable information on conformation (α and ν). This demonstrates that TF3 can resolve a remarkably narrower UHMW copolymer distribution, which would potentially be excluded or decomposed due to shear-stress in SEC.

The exponent from the direct relationship between *R*_G_ and *M*_W_ is defined by the scaling parameter “ν”, and the corresponding value of 0.64 (Figure 2b) is within the limits for a polymer that adopts a statistical coil conformation within a thermodynamic good solvent. Furthermore, the average *R*_G_/*R*_H_ ratio (ρ) of 1.71 is also within the limits for flexible polymer in good solvent, which further supports the interpretation of a moderately expanded coil. The term “α” is the exponent from MHS expression, which describes the direct relationship between [η] and *M*_W_; and α value of 0.76 in Figure 2c is supplementary evidence that the polymer behaves as a flexible coil (0.5–1.0) in a thermodynamic good solvent. The corresponding distribution in ρ (Figure 2f) and κ ratios (Figure 2e) for the UHMW SMAnh ranging from approximately 1.4–2.1 and 0.8–1.1, respectively, are closely comparable with empirical expectations.^13,16,28^

TGE-3DCoTF3 with quintuple-detection delivers a nondestructive and composition-sensitive route to resolve UHMW SMAnh copolymers across megadalton ranges while preserving coil integrity. The constancy of the *D*_T_ across all fractions confirms a uniform styrene-to-maleic anhydride ratio and validates a truly alternating copolymerization mechanism. This invariance establishes *D*_T_ as a quantitative, real-time indicator of copolymer composition, complementing spectroscopic methods.

The multidetector configuration enables simultaneous extraction of molar-mass, three independent radii, intrinsic viscosity, diffusion coefficient, and UV–vis spectra from a single experiment, providing a complete structural and compositional fingerprint. UV–vis responses clarify monomer conversion and reveal the optical origin of intense void-time signals due to molar-absorptivity and plug compression effects.

Comparative analysis of lower- and higher-UHMW copolymers differing by a 2-fold degree of polymerization, shows identical thermophoretic coefficients but divergent conformational behavior, indicating a transition toward a compact core–shell architecture at extreme chain lengths. Together, these insights confirm that TGE-3DCoTF3 surpasses conventional SEC and other FFF variants in resolving molar-mass, size, and compositional heterogeneity of fragile UHMW copolymers. The method establishes a quantitative benchmark for multidimensional polymer characterization and a foundation for probing structure–property relationships in advanced macromolecular systems.

## Supplementary Material



## Notes

(Section I) TGE-3DCoTF3 methodology: TF3 experimental setup, TGE separation program, theoretical background of TF3 analysis; and additional TF3/SEC Figures and Tables; (Section II) Copolymerization protocols: copolymerization procedures for styrene and maleic anhydride, ^1^H NMR spectra for conversion analysis, UV–vis spectra of monomers, and light-source calibration data (PDF)
