# Supplementary material for: Introduction of Temperature-Gradient Elution in Three-Dimensional Correlation Thermal Field-Flow Fractionation with Quintuple Detection for Monitoring Compositional Dynamics of Ultrahigh-Molar-Mass Poly(styrene-co-maleic anhydride)
Source: ACS Macro Lett. 2025 Dec 16;15(1):46–52. doi: 10.1021/acsmacrolett.5c00593 (PMC12825381; doi:10.1021/acsmacrolett.5c00593)
Supplement: Supplementary file 1 [file mz5c00593_si_001.pdf]

Supplementary Information:

Introduction of temperature-gradient-elution in 3-dimensional correlation thermal field-flow fractionation with quintuple-detection for monitoring compositional dynamics of ultra-high-molar-mass poly(styrene-*co*-maleic anhydride)

*Upenyu L. Muza,[a,b] * Michael-Phillip Smith,[b] Joshua T. Johani, [a,b] Bert Klumperman[b] and Albena Lederer,[a,b]*

[a] Department Advanced Macromolecular Structure Analysis, Leibniz-Institut für Polymerforschung Dresden e.V., Hohe Str. 6, D-01069 Dresden, Germany

[b] Stellenbosch University, Department of Chemistry and Polymer Science, Private Bag X1, Matieland 7602, South Africa

I. TGE-3DCoTF3 methodology

1. TF3 experimental setup

All TF3 experiments were performed on a TF2000 TF3 system (Postnova Analytics, Landsberg, Germany) fitted with a 270 mm × 20 mm stainless-steel channel and a 250 µm PTFE spacer. Tetrahydrofuran (THF) as carrier solvent was pumped at 0.30 mL·min^{-1} with an isocratic pump (PN 1130, Postnova). A recirculating chiller set to 5 °C stabilised the channel's cold wall at 15.7 °C, while Peltier heating maintained the hot wall at 104.8 °C, giving a nominal ΔT of 90 °C at 35.3 % heater power. The system operated at 4 bar pump pressure and 6 bar internal channel pressure, values well below the recommended 8 bar limit and sufficient to ensure stable parabolic flow. Samples were dissolved in THF to ≈ 8 mg·mL^{-1}, filtered through 0.45 µm PTFE membranes to prevent particulate carry-over that could foul the 250 µm channel, and introduced manually *via* a 100 µL PEEK loop that was over-filled to ensure complete flooding of the loop and inlet capillary. Samples were prepared and stored in brown, opaque screw-cap vials to minimise photodegradation.

2. TGE separation program

After a 60 s sample injection the 90 °C gradient was maintained for a 5 min focusing period, sharpening the injected sample against the cold-wall. The elution temperature-programme then proceeded through three sequential segments (Figure S1): (i) a 15 min isothermal hold at ΔT = 90 °C during which non-retained monomer and short oligomer species traversed the channel and appeared at the void time (t_0 ≈ 15 min); (ii) a linear ramp that decreased the gradient from 90 °C to 10 °C over the next 60 min, effecting size- and S_T-dependent separation of low- to UHMW copolymers; and (iii) a 60 min plateau at ΔT = 10 °C that fully eluted the largest, most thermophobic chains. At 140 min the power was switched off, collapsing the gradient to ΔT = 0 °C, and the channel was rinsed isothermally for 5 min at the original carrier flow-rate to clear any

strongly thermophoretic or partially gelled material; thereafter, back-pressure returned to baseline, signalling run completion. Throughout the run, the pump and channel pressures remained stable about 4 and 6 bar, respectively.

3. Quintuple-detection

Quintuple-detection has evolved to become the gold-standard for comprehensively characterizing the molecular blueprint and microstructural dynamics of eluting analytes as a function of multiple physicochemical properties. The following physicochemical property-to-detector relationships are primal in the advanced characterization of complex polymers – MALS: molar mass and R_G; DLS: D; and Visco: [η]. By applying various mathematical models and correlations as a function of these primal physicochemical properties (see SI Equations S1 – S8), a multitude of other molecular and structural properties are derivable; namely, R_H, viscosity radius (R_V), apparent density (ρ_{APP}), shape parameter (ρ), compactness parameter (κ) and their respective distributions.[1,2]

Eluent was monitored on-line by a quintuple-detector array comprising UV-Vis absorbance (Shimadzu**)**, Wyatt Technology detector suite (Santa Barbara, CA, USA)**:** a DAWN HELEOS II MALS photometer (18 angles, λ = 658 nm), an Optilab T-rEX differential refractometer, a ViscoStar III differential viscometer, and a Wyatt QELS in-flow DLS module integrated into the DAWN flow cell (back-scatter angle 173°) All signals were acquired with Postnova TF2000 software in combination with Wyatt ASTRA 8.0; and processed with its 3DCoTF3 routine, which synchronises retention time, spectroscopic contrast and physico-chemical observables to generate the data cube discussed in this work. Each specimen was analysed in triplicate. Absolute molar-mass calibration used a refractive-index increment *dn/dc* = 0.101 $mL \cdot g^{-1}$ for poly(styrene-*co*-maleic anhydride). MALS data were fitted by the Berry method after masking the two lowest

scattering angles to remove forward-scattering artefacts. MALS detector was normalised with toluene. The quintuple-detection signals are shown in Figure S1.

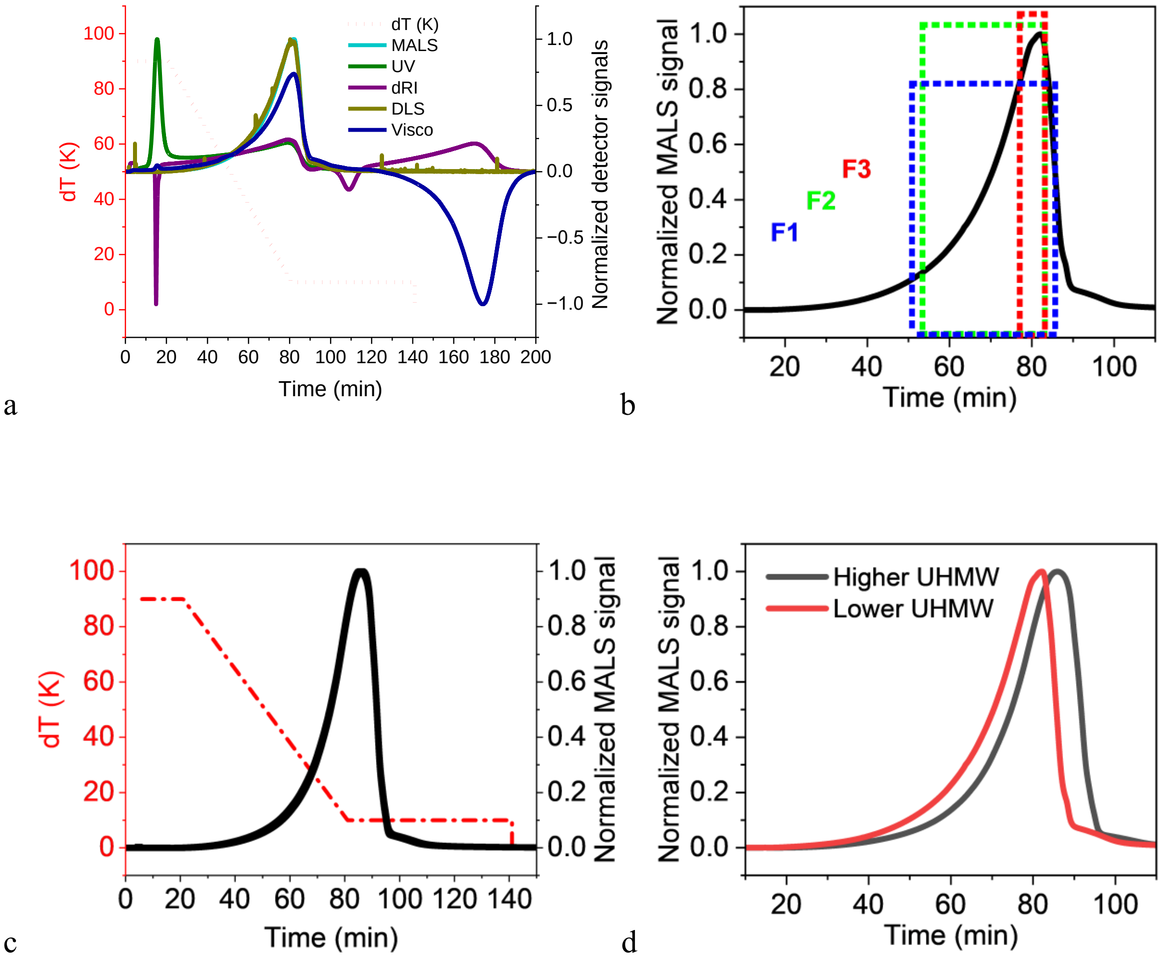

Figure S1. (a) TGE-3DCoTF3 with quintuple-detection run for *lower* UHMW copolymer: ΔT temperature-program (red dots, left axis) and normalized detector signals (right axis), respectively as a function of time (x-axis); MALS (dark cyan), UV-Vis at 254 nm (green), dRI (purple), DLS (dark yellow) and Visco (royal blue); (b) Three strategic fractions (F) for providing explicit leverage for probing how integration limits control the reported averages; (c) Normalized MALS signal (right axis) from TGE-3DCoTF3 of *higher* UHMW copolymer: ΔT temperature-program

(red dashed dots, left axis), as a function of time (x-axis); (d) Comparative analysis using normalized MALS signal for lower UHMW vs higher UHMW copolymers.

4. Basic theory supporting TF3 analysis

In TF3, the system void peak (t_0) contains the none-retained species, and this is where the none converted monomers and short oligomeric chains are expected to elute, owing to their low molecular size (Figure 1). The first feature in the fractogram is the void peak at ≈15 min, which also defines the channel's void volume, and therefore contains all species whose hydrodynamic diameters are too small to experience measurable thermophoretic retention. In TF3 theory, such non-retained analytes elute at t_0 by definition, consequential of the retention ratio $R = t_0/t_R$ collapsing to unit when the product of S_T and ΔT approaches zero, and this is typical for small macromolecules (below 10 nm Ø).[3]

For a polymer chain having N number of units, r_i as the position vector of the ith monomer and R as the center of mass of the polymer; R_G is defined as the root-mean-square distance of the monomer units from the center of mass of the polymer (R), and provides a measure of the polymer's size in solution (Equation 1). Using MALS detection, R_G can be measured from the angular dependency of scattered monochromatic light, and the size limit of measurement is typically 10 nm.[4]

$$R_G = \sqrt{\frac{1}{N}\sum_{i=1}^{N}(r_i - R)^2} \qquad (S1)$$

From the time-dependent fluctuations in the intensity of scattered monochromatic light, D can be directly measured from DLS using an autocorrelation function; and subsequently, R_H can be calculated indirectly by applying the Stokes-Einstein expression for D datasets (Equation 2): Where k is the Boltzmann constant, T is the absolute temperature, and η is the solvent viscosity.

$$R_H = \frac{k_B T}{6\pi\eta_0 D} \qquad (S2)$$

The [η] is a fundamental parameter that describes how a polymer increases the viscosity of a solution relative to the solvent's viscosity. It is defined as the limit of the reduced viscosity as the concentration of the polymer approaches zero. The viscosity radius R_V is derived from [η] using the following relationship, where M is the M_W of the polymer, and N_A is Avogadro's number:

$$R_V = \left(\frac{3[\eta]M}{10\pi N_A}\right)^{\frac{1}{3}} \qquad (S3)$$

As defined in Equations 4 and 5, the double-logarithmic plot of either M_W vs. R_G or M_W vs. [η] enables the estimation of plausible shapes and structural configurations for polymer chains in solution based on two analogous power laws, where M can be represented by M_W:

$$R_G = KM^{v} \qquad (S4)$$

$$[\eta] = KM^{\alpha} \qquad (S5)$$

The conformation of the polymer chain is defined by the two exponents α and ν, as a function of solvent quality. The conformation and MHS plots can be applied to derive complimentary information on conformation and microstructure as a function of molar mass, which can be represented by the weight-average molecular weight (M_w). The degree of compactness of a polymer is directly correlated to mechanical properties such as tensile strength, compressive strength, and hardness. Generally, highly dense and compact materials are usually stronger and more durable. From MALS data, the degree of compactness can be evaluated by calculating ρ_{APP} using Equation 6:

$$\rho_{APP} = \frac{3}{4\pi N_A}\frac{M_W}{R_G} \qquad (S6)$$

The shape factor (ρ) and compactness parameter (κ) are defined in Equations 8 and Equation 9, respectively; and they describe polymer geometry in solution independent of M_W. For a polymer dissolved in a thermodynamically good solvent, flexible linear chains adopt an expanded coil conformation as described by the respective RMS / R_H (ρ) and R_V / RMS (κ) ratios of approximately 1.5 – 1.9 and 0.9 − 1.0.

$$\rho = \frac{RMS}{R_H} \qquad (S7)$$

$$\kappa = \frac{R_\eta}{RMS} \qquad (S8)$$

II. Copolymerization protocols

Poly(styrene-*co*-maleic anhydride), (SMAnh) is produced in a wide variety of compositions (8 – 50 mol% MAnh) and molar masses ($10^3 < M_n < 10^5$ g/mol) and finds application from pigment dispersant to engineering plastic. Alternating SMAnh with ultrahigh molar mass ($M_n > 10^6$ g/mol) is rarely encountered, and its synthesis *via* radical polymerization virtually unexplored. A mechanistic study of ultrahigh molar mass SMAnh synthesis is currently underway, but in this contribution we already want to highlight some unexpected behaviour of the resulting copolymers in thermal field flow fractionation (TF3).

It is known that styrene and maleic anhydride possess a strong tendency towards forming an alternating copolymer. The 1:1 incorporation ratio of STY and MAnh moieties was confirmed *via* ^{1}H NMR spectroscopy, which agrees with this alternating placement of comonomers in the polymer chain. Homopolymerization of STY under identical light induced conditions led to considerably lower monomer conversions as compared to the STY/MAnh copolymerization (Table 2). For the STY homopolymerization, only oligomer formation took place. For the STY/MAnh copolymerization it is noted that the average molecular weights and overall conversions are dependent on the wavelength of the light utilized. These phenomena are under further investigation, but may be the result of variable radical flux as a function of wavelength.

1. Nuclear magnetic resonance analysis

Monomer conversions were determined by ^{1}H NMR spectroscopy (Bruker Ascend, 600 MHz, $CDCl_3$) by comparing the vinyl proton integrals of styrene and MAnh to those of trioxane; spectra were processed with MestReNova v 11.3.

2. Materials

All chemicals were purchased from Sigma Aldrich. Styrene was bought with 4-*tert*-butylcatechol as a stabilizer, >99% to inhibit monomer auto-polymerization.

3. Monomer Preparation

Styrene had its inhibitor removed 30 minutes before polymerization by passing the monomer through an aluminum oxide column three times. Maleic anhydride (MAnh) was purified *via* recrystallization in boiling distilled chloroform. The recrystallized MAnh was filtered and washed with cold distilled chloroform. The crystals were then dried over 2 days under vacuum at room temperature. The trioxane utilized as an internal standard was purified by vacuum sublimation at 45 °C overnight in a sealed Schlenk flask.

4. UV-Vis of monomer

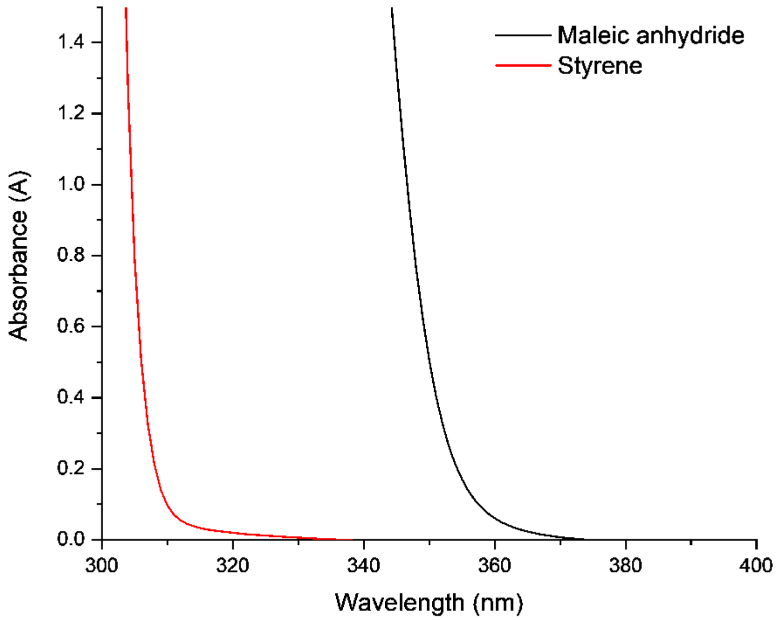

Figure S2. Batch UV-Vis spectra for styrene and maleic-anhydride monomers giving extinction coefficients at 254 nm and 340 nm.

5. Light sources

The lights utilized were calibrated with a Eurolite LM-50 Digital Lux Meter. The wavelengths were determined by a Thorlabs Photo meter. The LED strip utilized in this work (400 nm) was acquired from LEDs4Life (https://leds4life.co.za/) as (12 V, 14.4 W, 2 m). The 365 nm LED bulb utilized in this work was acquired from Bastion Paint (http://bastionpaint.co.za/).

Table S1: Light intensity determined by Lux meter.

**Light source**	**Light intensity for polymerization**
365 nm	1790 Lux
400 nm	5700 Lux

6. Light reactors

The light reactions were conducted in a temperature-controlled box at 25 °C. The light strip (400 nm) was wrapped inside a cylindrical PVC pipe. The 365 nm light bubble was held 5 cm away from the reaction vessel. The light sources were turned on 30 minutes before the reaction commenced to ensure all bulbs were at an equilibrated intensity.

7. Copolymerization of styrene and maleic anhydride:

To an oven-dried, clean scintillated vial (25 mL) equipped with a Teflon magnetic stirrer bar was added, styrene (0.25 g, 2.40 mmol, 1.00 eq.), maleic anhydride (0.24 g, 2.40 mmol, 1.00 eq.), trioxane (4.00 mg, 0.05 mmol, 0.02 eq.) as an internal standard, and dioxane (1.8 mL). The scintillated vial was then sealed with a rubber septum. The mixture was thereafter sparged for 30 minutes with argon. Once sparged, a t_0 sample was collected, thereafter the reaction was irradiated and stirred for 20 hours.

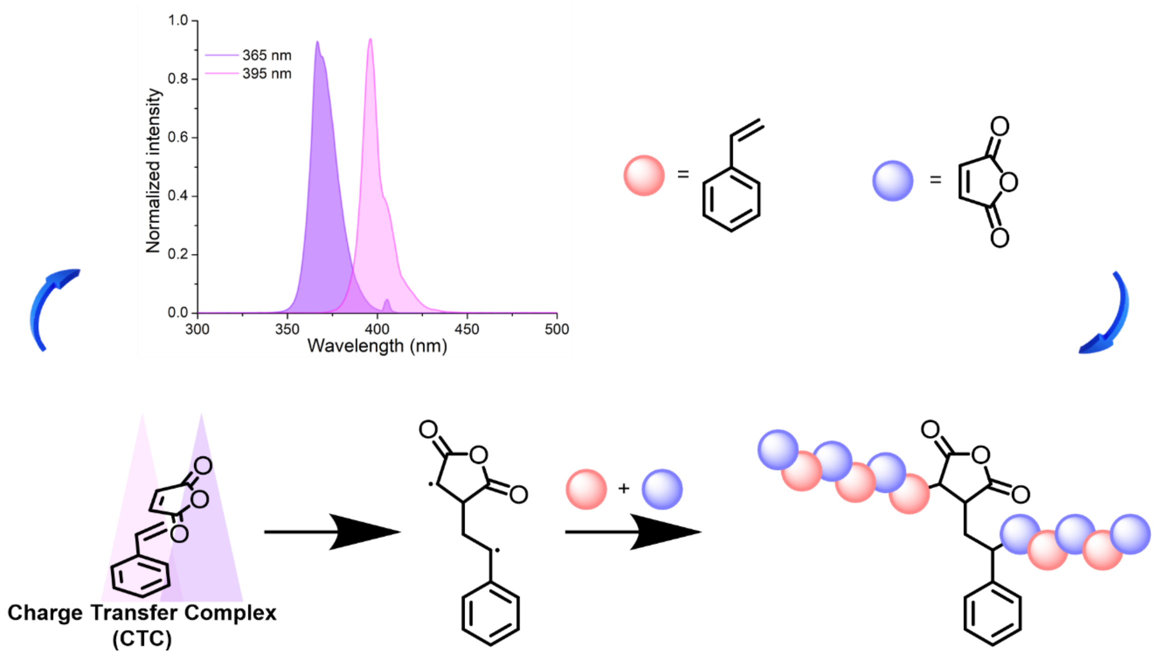

Scheme S1. Summarized biradical-photoinitiation copolymerization.

8. Preliminary copolymer characterization

Weight-average molar masses (M_n) and dispersities ($Đ$) were obtained by TF3 following standard calibration procedures; representative values after 20 h irradiation are summarized in Table S2 of the Supporting Information (e.g., 400 nm LED: M_n = 850 kg·mol^{-1}, $Đ$ = 1.29; 365 nm LED: M_n = 460 kg·mol^{-1}, $Đ$ = 1.46). All polymerizations and analyses were performed in triplicate; reported values represent the mean of the three runs, with deviations within ±5 %.

Table S2. Polymer molecular weight and conversions after 20 hours of polymerization. Determined by indicated analytical method.

Light source	MAnh utilized (g)	STY utilized (g)	MAnh conversion[b]	STY conversion[b]	M_n[a] (kg/mol)	*Đ*[a]
**365 nm**	0.23	0.25	99.3%	99.3%	460	1.46
**395 nm**	0.24	0.25	93.7%	97.8	525	1.56

[a] – determined by Thermal Field Flow Fractionation, [b] – determined by ^{1}H Nuclear Magnetic Resonance in $CDCl_3$

Reliable molar mass modelling was achievable using a broader integration window. Therefore, for molar mass determination only, results obtained with this extended window are reported in SI, as they provide a more representative estimate of the expected dispersity for radical polymerization.

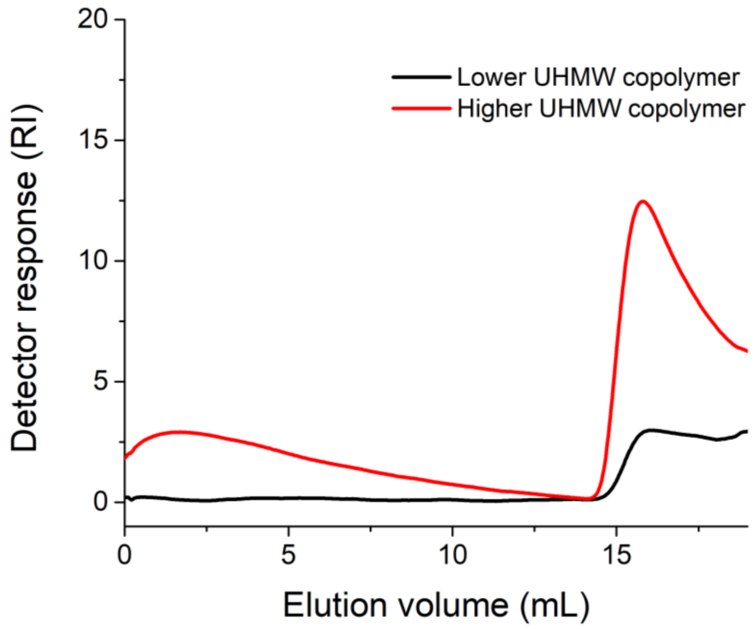

Figure S3. Conventional SEC fractograms showing unreliable and irreproducible data for the analysis of lower UHMW and higher UHMW copolymers.

9. Polymerization of styrene:

To an oven-dried, clean scintillated vial (25 mL) equipped with a Teflon magnetic stirrer bar was added, styrene (0.50 g, 4.80 mmol, 1.00 eq.), trioxane (4.00 mg, 0.05 mmol, 0.02 eq.) as an internal standard, and dioxane (1.80 mL). The scintillated vial was then sealed with a rubber septum. The mixture was thereafter sparged for 30 minutes with argon. Once sparged, a t_0 sample was collected, thereafter the reaction was irradiated and stirred for 20 hours.

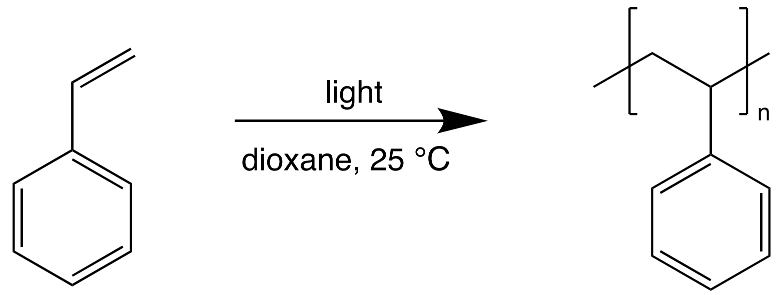

Scheme S2. Polymerization of styrene

Table S3. Polymer molecular weight and conversions after 20 hours of polymerization. Determined by indicated analytical method.

Light source	STY utilized (g)	STY Conversion[b,*]
**365 nm**	0.50 g	19.2%
**395 nm**	0.51 g	2.77%

[b] – determined by ^1H Nuclear Magnetic Resonance in $CDCl_3$, * - it was not possible to determine the molar masses of these polymerizations as the samples co-eluted with the system signal (~500 g/mol) in the Size Exclusion Chromatography system utilized

References

(1) Brewer, A. K.; Striegel, A. M. Characterizing the Size, Shape, and Compactness of a Polydisperse Prolate Ellipsoidal Particle via Quadruple-Detector Hydrodynamic Chromatography. *Analyst* **2011**, *136* (3), 515–519. https://doi.org/10.1039/c0an00738b.

(2) Muza, U. L.; Pasch, H. Thermal Field-Flow Fractionation with Quintuple Detection for the Comprehensive Analysis of Complex Polymers. *Anal Chem* **2019**, *91*, 6926–6933. https://doi.org/10.1021/acs.analchem.9b01384.

(3) Burchard, W. Static and Dynamic Light Scattering from Branched Polymers and Biopolymers. In *Light Scattering from Polymers*; Springer Berlin Heidelberg: Berlin, Heidelberg, 1983; pp 1–124.

(4) Podzimek, S.; Vlcek, T.; Johann, C. *Characterization of Branched Polymers by Size Exclusion Chromatography Coupled with Multiangle Light Scattering Detector*. *I. Size Exclusion Chromatography Elution Behavior of Branched Polymers*; 2001.
